# Treatment of Pleurisy and Empyema

**Published:** 1878-08

**Authors:** 


### On the Treatment of Pleurisy and Empyema.

At a meeting of the London Medical Society, in April, after detailing several cases, Dr. D. M. Williams said he would not occupy time in recording the histories of cases of pleurisy with serous effusion, but only remarked that they usually took from six week to six months' time before the fluid was absorbed, if it had been thrown out in any considerable quantity; but, notwithstanding the longest time (three months), such cases have done well in his practice, the lung expanding once more. He felt, therefore, encouraged to treat these with diuretics, iodine, etc. Nevertheless, as an illness of two or three months was often a very serious matter to the patient, sometimes causing the loss of employment, or of business, he had asked himself if there was no way by which they could hinder the effusion of serum, or at least, its accumulating in any great quantity. When they remembered that the sharp stitch in the side in pleurisy, caused by the friction of the two surfaces, was intended to limit the movement of the lungs, we could not but feel that nature had already given them the hint—stop respiratory movements and the mischief is prevented. They had already recognised that principle in peritonitis, synovitis, etc. Here, however, they had to do with an organ whose function could not be stopped with safety to the patient; but he thought they could sufficiently limit the movements of one lung so as to hinder the formation of, at any rate, a large quantity of fluid in the chest, and this he had attempted to do by strapping the inflamed side firmly with plaster, taking care to bring the ends of the plaster to the opposite side of the spine and sternum. The first result of such treatment was a great relief from pain; the next was a subsidence of fever, etc. To say that a few cases had been thus treated successfully was to prove nothing, as it might be reasonably urged that these were slight ones, or only dry pleurisy; a medical friend, however, had, at his request, already treated eight successive cases successfully. Having detailed one case treated successfully in that way, Dr. Williams said that since writing the description of that case he had heard that an American surgeon had claimed all thoracic diseases for the domain of surgery, and treated all such by pressure, but not exactly strapping, while Dr. Fred. Roberts had, he found, already advocating strapping. Again, with reference to empyema, he wished to dwell especially on the condition of the lung after recovery. It is pretty generally admitted that in children under twelve years of age, such cases usually ended well, the lung expanding again, whatever may have been the amount of mischief; while in adults this was rarely the case, the lung being almost always so damaged as to be unable to support life alone. This is surely not a cure in which we can take much comfort, however brilliant the recovery might appear to the patient and his friends. On the contrary, they should not consider such cases cured unless the lung once more occupied its normal situation. He had asked himself the reason why the lung should expand so easily in children, and scarcely ever do so completely in adults; and he thought the condition of the ribs in children explained it. Thus, in children the ribs yielded rather readily to the atmospheric pressure without losing their spring; consequently, there was not left the same amount of empty space in the chest when the fluid was removed as in an adult, while, at the same time, there was a constant suction power from the tendency the ribs had to resume their natural curve. In the adult the ribs yielded much less readily, but eventually became bent in permanently. Now, it had occurred to him that if they could imitate this, and find some plan of keeping the pleural cavity more or less constantly in a state of vacuum before the ribs bent, without using injurious force, they would help the lung to expand; and for this purpose he had invented a cannula with a very delicate valve, which, by acting only one way, allows fluid or air to escape from the chest, but instantly hinders air entering it. Now admitting that a patient, with this tube in situ, coughs only twice each hour, the force of coughing easily expels fluid out through this tube, while the valve effectually hinders the entrance of air into the pleural cavity. Thus more or less of a vacuum is formed, and in proportion to the amount of vacuum was the aid given to the air rushing through the trachea to expand the lung. The cannula had been purposely made short, in order that it might be worn until the lung expands pretty freely. It is secured in its position by tapes passed through the slits on either side. Seeing, then, that pleurisy, if detected at the commencement, may be greatly relieved, and effusion to any any great extent hindered, by firm strapping, it became exceedingly important to detect it early. There was, of course, no difficulty in doing this when effusion had already taken place, but previous to that he considered it no easy matter to do so, in children. The pain in the side and friction sound, upon which they depended so much in the adult, were of little use in dealing with children, and he had adopted the following plan:—Suppose a child two years old; very cross and feverish, with a slight cough, and these symptoms not explained by any other disease. He wrapped the child loosely in a blanket, and having put it sitting on its mother's knee, waited a minute or two; presently it coughed, and immediately cried. Then he put it sitting upright, and watched carefully; next time it coughed they would notice that it shrank to one side. He placed a warm flannel on that side, and let the mother keep up firm pressure; the child coughed again, but the crying and shrinking were less; and if on very careful examination there was slight dullness, and respiration a little feeble, without crepitation, they might be sure that they had a case of pleurisy; and if that side be strapped the child would probably have a good night, and be much better next morning. Supposing effusion to have already taken place (and the patient be a child), the fluid should be removed at once if the constitutional disturbance is great, as in all probability it is purulent, serous effusions giving comparatively little indication of their presence unless the quantity of fluid is great; indeed, he had been often surprised at the small amount of constitutional disturbance where he had found a large quantity of fluid, in both children and adults. If a doubt existed, the needle of the hypodermic syringe might be used to explore, as recommended by Dr. Barlow and Mr. Parker; but in an adult he would seek to be guided entirely by the constitutional disturbance, which, he thought, was much greater where the effusion was purulent. If, in a child, a fistulous communication with a bronchial tube had already occurred, and pus was being freely expectorated, he would first strap the side and wait a few days, as that seemed to be Nature's best and most successful mode of removing pus from the cavity of the pleura (while fistulæ through the thoracic parietes almost always ended badly). Should the relief be insufficient, or the strength giving way, he would then tap the side; but, in the case of an adult, he would tap at once, notwithstanding that a fistula had occured, as he had not seen a single such case do well without tapping. The pleural cavity should be freely washed out with a weak solution of carbolic or sulphurous acid, or iodine, and if, after a week or two, the pus remained fetid, the wound should be enlarged with a probe-pointed bistoury, in order to give exit to any pieces of false membrane or clots of blood which might be decomposing.—*Half-Yearly Compendium.*

